# Making things specific: towards an anthropology of everyday ethics in healthcare

**DOI:** 10.1007/s11019-024-10204-z

**Published:** 2024-05-01

**Authors:** Jeannette Pols

**Affiliations:** 1 Department of Ethics, Law & Humanities, UMC Amsterdam, Amsterdam, The Netherlands; 2 Department of Anthropology, University of Amsterdam, Nieuwe Achtergracht, 166, 1018 WV Amsterdam, The Netherlands

**Keywords:** Ethics, Philosophy of the good life, Ethnography, Everyday values, Chronic disease, Clinical knowledge, Practical knowledge

## Abstract

This paper is the English translation and adaptation of my inaugural lecture in Amsterdam for the Chair Anthropology of Everyday Ethics in Health Care. I argue that the challenges in health care may look daunting and unsolvable in their scale and complexity, but that it helps to consider these problems in their specificity, while accepting that some problems may not be solved but have become chronic. The paper provides reflections on how to develop a scientific approach that does not aim to eradicate bad things but explores ways in which to live with them. Crucial in this quest is the attention to how we conceptualize problems, and whether this is specific enough for addressing present day concerns. I propose an anthropology of everyday ethics as a way to study people’s everyday ways of handling a variety of goods in practice. I draw specific attention to exploring aesthetic values in everyday life amongst these, values that are used abundantly to qualify events in everyday life but rarely theorized in philosophy or social science.

## Big problems in health care

There are great worries and nightmare scenarios about health care, globally, but also in the Netherlands, which is the situation from where I am writing. There are great concerns about a steadily growing aging population, while the number of young people to provide care decreases. We witnessed decennia of high hopes that technology would provide solutions by making care more efficient and stimulating self-management by patients, but these hopes stubbornly refuse to become reality, even if they are still one of the driving forces of Dutch health care policy (Rijksoverheid [Dutch Government] 2022). The challenges did not stimulate a movement of solidarity to deal with them, but instead nurses and other health care workers such as general practitioners are increasingly leaving health care and there are staff shortages (Groot and Schaaijk 2023). Citizens increasingly fear that they will not be able to pay high insurance and health care costs, and they develop more and multiple chronic diseases, while preventive strategies hit a brick wall because of poverty, marginalization, distrust, and digital and other forms of illiteracy (RIVM [National Institute for Public Health and the Environment] 2013; Wonderen and Peeters 2022). Neoliberal attempts to organize care around efficiency and cost reduction increasingly show their downsides with increased bureaucratization, low quality of care and inaccessibility of care (RVS [Advisory Board for the Ministery of Health] 2023). Cost control has increasingly been the answer, rather than asking questions about what we actually want to achieve with health care, and what we find of value.

These are Big Problems, described in general terms. In this paper I argue that it helps to make Big Problems *more specific*. To zoom in and focus, hence analysing in detail what the problems are. Paradoxically, it might help not to think that we can *solve* Big Problems, but rather that we have to learn how to live with them. The older people will be there. Nurses will keep looking for satisfactory working conditions. Technologies will never solve all the problems we have, and will also always create some extra ones. It is comparable to the other Big Problems we are facing these days: they will not go away. Climate change, people on the move in the hope of escaping deprivation, death and violence, and the challenges of feeding all of Earth’s inhabitants and providing them with fresh water; these are all problems that are there to stay.

This is, I propose, and I will restrict myself to health care for this paper, not something to merely despair about, but demands that we try to figure out ways to best live with these problems. From this perspective, chronic problems may be an inspiration to find new ways of addressing the global issues that concern us. For this, we need to understand that the ways in which we speak about problems, and the ways in which we research them, are crucial for finding ways to address them. Finding tools to reconceptualize problems or concerns, by specifying them, may help to change the ways in which we try to do something about the situation.^1^

What we need for this, I suggest, are research methods to study specificity. Our scientific traditions are good at studying Big Problems. Epidemiology aim﻿s to generalize results over large populations, Big Data, research with ever larger datasets, is increasingly popular.^2^ Academic ethics are good at solving big and abstract ethical problems, such as who gets priority at the Emergency Unit in times of a pandemic, when resources are scarce (Verweij et al. 2020), or ethical problems at the beginning and end of life. This is important work. What I want to add to this is an empirical ethics of specificity: the anthropology of everyday ethics.^3^

I ask:

1. What does an anthropology of everyday ethics that studies the values of everyday life entail?
2. How does the understanding of aesthetic values as a form of everyday values clarify what is at stake in care practices for people with chronic disease?
3. How might an insight into everyday life values help dealing with health care problems we are facing?

## What is everyday ethics?

An anthropology of everyday ethics is concerned with the empirical analysis of our everyday dealings with values. Every day, at home and at work, we make many little decisions about what we will do, informed by ideas on why this would be good.^4^ Or we are subtly urged to do things in certain ways, by various pressures to respond to emails or phone messages, or by apps that tell us we have not made enough steps in our day. For many decisions we have developed routines, like brushing our teeth every day, and taking the kids to school. Values are also embedded in our technologies that tell us whether what they measure is good, or demand action. These are, one could say, solidified ways of doing good things without having to reflect every time on whether we should do these things or not. Everyday values, their guises, activities, relations and evocations, are the subject matter of the anthropology of everyday ethics. The anthropology of everyday ethics does not, at least not in the first instance, *judge* this work with and of everyday values, but *studies* it empirically. What do people and machines make important, and how does that influence what they are doing? What are the effects of their strivings?

### A case to think with

To illustrate this and honor my own call for specificity, I start with a case.

Carola has type 1 diabetes. Since she was 11 years old, on a daily basis she has measured her blood sugar levels and injected insulin because her body does not provide it. The aim is to keep blood sugar levels from moving to extremes: not too high in order to prevent longer-term complications; and in the short term not too low, because this brings the more immediate risk of a ‘hypo’ that can lead to unconsciousness. There are many technologies for measuring blood sugar levels on the market, and Carola uses an insulin injection pen for her injections. She measures her blood sugar levels five times a day, taking a drop of blood from her finger, and decides on how much insulin to inject. With regular appointments, her GP checks her measurements. Carola gets quite upset at one of these appointments:

You go to your GP and he looks at the measurements and starts: “Well, you should be careful, this should be lower.” And then you get the whole sermon. And then I think: you should try this, to pay attention to this 24 hours a day! I am not a machine! I *know* that these measurements are not good… And then I start (strategically) timing my measurements, so I don’t get lectured.

In the past, Carola has used insulin pumps that measure blood sugar constantly and inject insulin when necessary. But Carola is certain about it: she does not want to do this anymore. She says that it made her stressed, because it made her check her blood sugar too often. You do not have to prick your finger with a lancet several times per day to check your blood sugar levels (because the pump is inserted on other places of the body, most often the upper arm or belly), but the pump still made her check more often. ‘When I was young, I would measure twice, maybe once more before going to sleep. I would inject some insulin and add some in the early evening. That way you are much more relaxed. If you feel well and measure less, the disease is less central in your life.’

Carola passionately speaks about her work and about playing the clarinet. She says she would rather spend her time on playing than on her diabetes. ‘Of course, you have to pay attention to the diabetes, it cannot be avoided. It’s ok. But then as little as possible…’

When reading this story one might think: well, this is not very nice, to have a chronic disease like diabetes; you have to think about it and handle it, you have to pierce your skin often, and you have to deal with GPs that may lecture you. But you might not make an association with *ethics*, since there are no Big Dilemma’s here, nor are there principles at stake. However, there *is* food for ethicists here: for ethicists who are concerned with everyday ethics. I will explain this.

In Carola’s story there are many values or ‘goods’, even if they are not all moral values or goods. This is of interest to researchers who investigate the ethics of everyday life, that looks at the variations of values and their relationships in everyday practices.^5^ In Carola’s story there is the bad of hypos and piercings, and there are good glucose levels when they are stable. There is the nagging GP who does not help and upsets Carola. There are the clinical fashions of regulating more tightly or in more lenient ways. These fashions that relate to the use of technologies make some goods central but ignore others. For example, the centrality of the disease in everyday life that comes with constant measuring is in tension with the more easy-going life of measuring less frequently. There are trade-offs between care for diabetes and the pursuit of certain passions, such as work and music. ‘I am not a machine’, says Carola. Such an iron discipline would rob life of its freedom and spontaneity.

These are not trivial matters: the anthropology of everyday ethics asks how people attempt to do this, to live a life that is as good as possible. It is about persistent everyday work with the different kinds of things we find important. How do people attempt to live a good life, what is it that they care about when doing so, what helps and what makes this complicated? How do objects like insulin pumps or clarinets support this work—or operate in tension with it?^6^

Everyday ethics is hence about everyday work with values or ‘goods’, with things we find important. This can be about all practices and moments where values are at stake, and when questions emerge as to what is good, beautiful, and true (or healthy, efficient, juridically sound, scientifically shrouded, practical, climate friendly, and so on). Rather than being clearly separable, such values often come together. What is proclaimed as being ethically sound, also has a claim to be true, and is presented in a particular style. The work of studying everyday values does hence not only concern moral values, but consists of studying the constant weighing-up of goods, and the question of how this is done -for better or for worse. These everyday forms of good are always concrete and specific for a situation. There may be general rules and guidelines to help to deal with such problems in health care. But even then, these have to be translated to and weighed within a particular context. What is important for *this* patient, *here* and *now*? How do caregivers and patients relate to such questions?

## The setting: problems that do not go away

My particular concern is with everyday ethics in care for people whose disease or disability does not go away. Everyday ethics are also important in curative care and acute care, because there is also a striving for care that fits a particular patient, even if there are more protocols for it, and clearer end-points to aim for. However, in care for chronic diseases it becomes very clear that the persistent juggling of values will never come to a final conclusion. The ‘bad’ of a disease will not go away. Its manifestations will change, symptoms will shift, and new situations will demand new solutions. Hence, patients and clinicians will continue to look for the best possible options in specific situations. It is part of the clinical knowledge and skills of professionals and the practical knowledge of patients (Benner 1984; Pols 2014). They try to figure out how a life with chronic disease can best be lived from one situation to the next. There is never a clear and general end point to decide on this, as the types of problems and possible solutions shift. ‘As good as possible’ can mean very different things at very different moments. The result of this persistent ‘tinkering’ is often difficult to predict—as with some blood sugar measurements (Mol et al. 2010).

Amongst the many variations of values as well as the relations between these values in the lives of people with chronic disease, the question becomes what can good care and a good life be with disease. The good life is not a faraway ideal, or a set of prescriptions to live by or to be learned from famous patients who show how to do this. It is about everyday life, about mundane things, about everyday goods and bads and ways to deal with these.

Everyday *aesthetic* values become important here. ‘Aesthetic values’ is a sensitizing concept to empirically detect and specify values and conventions that motivate people and invites likeminded people to relate to each other. First, aesthetic values refer to what people find motivating, proper, fitting, or nice in terms of the ways to shape everyday life (Pols 2019). If continuous measurement is a way to regulate diabetes, but also makes the disease more central in life, is the desirability of an insulin pump then an aesthetic or a moral question?^7^ Or can it be both? Is a good life also a beautiful or pleasant life? And if this is the case, how may we think about such aesthetics, and how may that help to shape and evaluate lives as good or not so good? Second, aesthetic values refer to the creative work of organising everyday life. These matters relate to local conventions rather than general ideals or abstract concerns. Aesthetic conventions may be more or less solidified or visible to those living them.

The anthropology of everyday ethics aims to create academic space, concepts, techniques and methods to practice and think about everyday values, and the consequences of the fact that people are so busy with organizing them. Aesthetic values and the creative work to live life as well as possible need more academic work and the development of fitting concepts and methods to study them.

## Everyday life values in health care research, or the creativity of methods

The aesthetic metaphors also point to the active creation of worlds through scientific work.^8^ Here, scientists have something to learn from the arts.^9^ To study care practices for chronic disease new methods to study specificities were needed.^10^ Traditionally, the sciences use metaphors of ‘discovery’ or ‘establishing the facts’. The idea is that the scientist makes ‘nature speak’ (Rorty 1979). However, the use of different research methods shapes in important ways what kinds of results will be shown in research. A microscope reveals different things than a pair of binoculars. It matters *how* the world is made visible, what techniques and methods scientists use to do this.^11^ This is why scientists could learn from the world of arts. Scientific work also *creates* things. CRISPR-Cas gene editing, cutting parts out of genes to change the nature of the being that is to be born, is a clear example of how scientists also create things. But with less dramatic techniques like vaccinations, the sciences also interfere in people’s lives. And even more mundane, science and technology have an impact on how we measure blood glucose levels and brush our teeth. Hence it makes sense to reflect on how the sciences make things visible, and how this also shapes the relationships between the makers and receivers of scientific interventions.

If I want to study everyday ethics, the question arises about what scientific practices and concepts would make such research possible. Everyday scientific practices, the techniques and the concepts used, but also the way in which researchers relate to one another and to their research subjects, allow for the study of some things and not others. In my career, the question has been how to study long term or chronic care practices, and how to evaluate these practices. The methods at hand in ethics, medicine or the social sciences were not a good fit for this. We needed new methods to study how people attempt to do good things or give good care. What values are important here? What words and things influence the attempts to realize them? What are the effects of these strivings? And is it possible to improve or support all of this ‘doing good’?

## The study of health care practices

The attention to specificity in qualitative ethnographic research fits the clinical knowledge of professionals, and the practical knowledge of patients (Pols 2014). Clinicians spend 99 per cent of their time on patient care, implying the examination of what is needed for *this* patient at *this* moment. Medical research, however, is mostly concerned with generalizations. Generalisable knowledge is, of course, important, for instance because it will increase the probability that the medication we take will help us. Clinical knowledge is concerned with specific cases on an everyday basis. This form of knowledge is taught ‘at the bedside’ to novice nurses and doctors. But this does not mean that such knowledge is only valid in one particular situation. Clinical knowledge concerns the experience and skills of clinicians in the sense of having experience with something, and having learned something from things seen before. For example, once felt, a clinician is able to feel a ‘swollen liver’ or sense that ‘something is wrong’ with the patient who might present with common enough complaints. This sense of something not being quite right is embedded in a form of clinical reasoning and experience, conscious or not. There is work on clinical reasoning and there is a tradition in medicine to learn from clinical case histories. This often concerns diagnostics. Everyday ethics research can support the articulation of clinical knowledge and help to find wider applications in clinical practice.

## Using ethnographic methods

To connect research in medicine with research into clinical practice, proper methods are needed. Ideal candidates to study specificity are the ethnographic methods used in anthropology to study the specific and the everyday.^12^ Anthropologists often do ethnography far away from their home turf, where the everyday is quite different from what they are used to. But the study of everyday life and its specificities can also be done in the global north.^13^ The kind of anthropology I engage in is the study of practices and materiality.^14^ It concerns the study of the local relationships between words, things like blood sugar measuring devices, and what people do. I like to call this a *generative hermeneutic object* science. *Generative* because it wants to support the fruitful interpretation of problems that are relevant today. *Hermeneutic* because it is a science that aims for interpretation. And *object* science because it does not centre around methods, as in the quantitative sciences. There is a lot of care for the creation of methods there, methods that are good and standardized, mostly with the aim of filtering out interests, bias and error.^15^ These methods are normative, they are oriented towards creating good scientific knowledge, with objectivity as an ideal.

In the sciences concerned with specificity, the object sciences, the object of research is the leading factor, and methods are adapted to the specific object one wants to learn about. What methods would help learning about it? The collection of statistics or open questions? A focus group discussion? Or is it better to participate in someone’s everyday life for a while?^16^ The ideal is not to make the researcher ‘disappear’, but to make explicit how the method and the positionality of the researcher impact the object of research, and what this reveals about that object. The researcher needs to justify the concepts and techniques used, because they are co-responsible for what results will emerge. What does a good life with a chronic disease look like, and how can it be studied best? And how can this be done if language and cognitions are not the best entry point, such as doing research with people with dementia (Driessen 2023), learning disabilities (Dronkert 2023), or severe psychiatric disorders (Pols 2023a, b; Muusse et al. 2020). Can they be included in research in meaningful ways? What research practice would fit their lives? The anthropology of everyday ethics is well equipped for exploring these questions, because it does not solely rely on verbal exchanges, but observes what people *do* and how these doings show their appreciations (Pols 2005).

## Aesthetic values to study the good life

To study the everyday lives of people with chronic disease as good lives that also include bad things, I propose to learn from an old philosophical tradition that was concerned with the quest for truth and wisdom, and linked this quest to everyday ethical and aesthetic values (Hadot 2004). Everyday aesthetic values are values that are linked to conventions of what we find pleasant, nice or appropriate. The study of aesthetic values in everyday life is a good example of how things tend to be forgotten if we have no concepts to talk about them, or methods to study them. In the modernist neoliberal times in which we live, aesthetic values or beauty are seen as matters of taste (Pols 2019). It does not make much sense to dispute aesthetic preferences. Everybody should be free to pursue their own particular tastes, be this punk music, folk songs, ballet or finger painting. The task for the neoliberal state is to guarantee individual freedom for people to strive for their own ideas about what is beautiful. The state does not interfere with the nature of this beauty. Or rather more precisely: there are many fights over definitions to demarcate what is a matter of individual taste, and what should be regulated by the state; think of veils or face masks. Are these matters of good taste, religion, or rights? Or health, and what kind of value are we talking about then?

We lack a *scientific* language to conceptualize everyday aesthetic values, and particularly their social importance. Aesthetic values and their social functions are difficult—or impossible—to theorize within the idea of beauty being a matter of individual taste. Everyday aesthetic values are also difficult to study with a form of ethics that is concerned with universal principles. That we lack an idea of the social dimension of aesthetic values is not without risk. One consequence of this was that there was little protest when the cultural sector was shut down during the COVID-19 pandemic. The Dutch health minister advised people to ‘watch a DVD at home’ instead. Nothing essential seemed to be lost.

So there is a scientific blank, but in everyday lives aesthetic values are ubiquitous, once you start paying attention to them. We speak of a beautiful death, an appropriate gift, and a wonderful goal. There are friendly nurses and fitting ceremonies. There are even rule-based aesthetics of everyday life and how to relate to other people: etiquette. A normative etiquette that tells one how to behave may seem a bit outdated in the era of the individual. However, people keep creating very different styles of relating to each other, while excluding other styles and people. Take for example the ceremony in the Netherlands to inaugurate a new professor. The established professors attend the ceremony in black robes and hats. This is not merely about rituals that are nice and entertaining for the audience to look at. It links aesthetic matters with important hierarchies as to who may speak and who should be silent, and who may promote others. Aesthetic qualifications and styles of organizing things are not mere matters of personal taste, but are important for organizing forms of living together. In academia these forms are also contested as being too hierarchical every now and then. They point to ways of understanding what is true, proper, and good. The dress code and ceremonies in Dutch academia have not been contested recently, even if the hierarchical organization of (Dutch) academia is being debated with a call for more diversity and less hierarchy.

The example of the academic robes shows that aesthetic qualifications and ways of organizing things are not mere matters of individual taste and preference, or of exotic forms to relate to one another. Aesthetic qualifications are creations to create coherences in society and help to understand these coherences as solidified everyday values or aesthetic conventions. They relate to the ways we understand what is true and good. Different from the concerns with aesthetic values in everyday lives of people with chronic disease, rule based aesthetics put aesthetic concerns at the centre.

## Everyday workings of aesthetic values

Aesthetic values are connected to the senses that register something as nice or not so nice to look at, smell, hear, and so on. This makes aesthetic values—like other everyday values—difficult to define in abstract terms, but great objects to study empirically. Aesthetic values work differently to the principles that motivate rules and laws. Rules, norms and laws prescribe or prohibit things, whereas aesthetic values are *motivating*.^17^ They refer to conventions, by following, criticizing, or re-designing these. This does not relate to the ‘most individual expression of the most individual emotion’, which would be a modernist understanding of what art is, but relates to desires we share with others. Aesthetic values are not embraced through rational arguments, but by what people find motivating and what appeals to them.

Another example of the social character of aesthetic values is hippy culture. Hippy culture showed aesthetic characteristics that were different from what was considered appropriate by the post-war generation. Think of long hair, flowers, campfires with a guitar and smoking marijuana, free sexual morals and driving a deux chevaux. Simultaneously, these colourful evocations came with critical ideas about democracy and middle-class morality. The hippies argued for new ways to govern society. They wanted more participation of citizens in governance and more freedom in life, and they practiced this where they could. Critical (feminist) analyses of the hippy movement’s ideas are also well known. But in whichever way one might judge the hippy culture, hippies are an example of the connection between aesthetic forms, political proposals, and ways of understanding things.

Aesthetic forms are, therefore, much more important than the idea of individual taste would suggest. Aesthetic values hence are far more interesting than merely depicting personal taste. They are motivating social values that influence what people do because certain aesthetics appeal to them, not because these values can be rationally argued for or regulated, but because they involve a motivation or seduction to practice a certain lifestyle, and to follow, criticize or re-design dominant conventions.

## Ancient Greece

The study of everyday ethics and aesthetics can learn from traditions in ancient Greece and the pre-modern humanistic philosophers that drew upon these traditions. Pierre Hadot (2004) describes how these philosophers tried to live philosophy *as a practice*, as a way to shape and examine a good life and to teach others about it. They were not seeking to produce abstract theory; rather, they wanted to study and improve everyday life by inquiring after wisdom about ways to live. They educated their contemporary citizens by questioning them about what they were doing and why. Socrates is an exemplary character here. Philosophy as practice demanded courage to speak up. Socrates was put to death because he ‘spoilt the youth’.

Foucault (2011) describes how the Cynics ‘lived as dogs’, barking at their fellow citizens and shocking them by their disregard for anything conventional, and by abstaining from luxury and fame. They believed that anything *natural* was good, true and beautiful, including living in a barrel and defaecating in the streets. These practices, one can imagine, were very much against the value that most Greek citizens attached to reputation, fame and wealth. The Cynics were quite an extreme example of a philosophical practice; the Stoics, for instance, were more moderate.

The practical examinations and reflections of the philosophy-as-practice philosophers concerned the everyday lives of their contemporaries, the citizens who lived among them. By examination and practice, people were urged to improve their ‘ethos’ or way of living a good life. The philosophers demonstrated how to turn everyday life into a work of art, by living in ways they understood as good and true, and developing these ways by examining them and putting them to the test. What was good, true, and beautiful hung together. The idea of truth came with the idea of what was good and proper to do. The philosophy-as-practice philosophers prioritized their practices over writing up ideas, and some of them, Socrates for example, died without leaving anything in writing. This may be a reason why the tradition has been largely forgotten.

## Everyday values in health care

Why is this almost forgotten philosophical tradition important to medicine? First, it reminds us to not forget about the history of doing things in certain ways. In medicine, it is good to remember the clinical fashions of regulating type I diabetes, in order to reflect on the different consequences of doing this in one way or another. Another example of the importance of knowing about clinical history is psychiatry’s earlier concerns about ‘treating homosexuality’ as a mental illness, rather than supporting people to live well with their sexual orientations. The Dutch Society for Psychiatry has since apologized for these views and related practices. The history of clinical practice is an important source to learn from.

But philosophy-as-practice can also be applied to help learning from the present. To this end, it needs some modernization. Again, we need an empirical turn, away from normative prescriptions on what to do, towards an empirical analysis of the workings of everyday values in everyday care practice.^18^ The analysis would not start with a judgement about what is good health care or a good life with disease—what these *should be*. The first step is to see how people already give shape to what they find good, and how the use of words and things helps or hinders them in their attempts. What is of importance here? What are the effects of cherishing certain values? Can values in tension be reconciled? When people live with a chronic disease, an ideal life might never be achieved. A good life means dealing with the problems and questions that are a part of it. The good life is never ‘fixed’ or achieved, but is always under construction. The striving is for *as good as possible*, while realizing that this is also often difficult. The question is, again and again, what is important or of value in the here and now. These are precisely the questions a clinician would ask when talking to their patients.

Clinical work is a nice metaphor for the everyday striving for something good *and* for the fallibility of these attempts. Clinicians are masters in practicing everyday ethics and aesthetics. They try to figure out what is good to do, or least bad, for every patient, again and again, in always specific situations. The core of clinical work is to figure out in an informed way what is the best or least bad way of living with disease for any patient they encounter. I imagine nurses to be exemplary figures in the practice of everyday ethics. Nurses co-examine and co-shape the everyday goodness, beauty and truth in the lives of their patients.

## Sniffer dog ethics

How can an anthropology of everyday ethics contribute to care work in practice? I showed how it studies what motivates people and what is important to them. Such research fits clinical work, with its knowledge about and handling of specificities, everyday values, and about problems that do not go away, but have to be tinkered with without guarantee of success. It does not look for abstract rules or general answers, but seeks to pose the question: what is needed here and now, as an intervention in everyday life?

This does not fit with traditional ‘watchdog ethics’. We need watchdog ethics, because they formulate criteria for what to do in complex matters. But for the work with everyday values, the metaphor of ‘sniffer dog ethics’ might be more fitting.^19^ The sniffer dog ethicists trace and analyse values in all their variety in everyday practices. To learn what values matter where, which ones run into trouble and which ones provide chances and possibilities for a good life.

A good example is the study of technologies in healthcare. We have already learned that technologies are difficult to evaluate with clinical trials, the methods invented for the study of medication. Different from taking a pill, the introduction of new technologies changes forms of collaboration in a practice. Nurses, doctors, patients, technicians, they all start to do different things. This makes it hard to see if the evaluation shows the effects of the technology, or of these new collaborations.

Sniffer dog ethics can then help by examining closely what values come to matter in the practice of using new technologies, what problems are solved and what problems are ignored with the new forms of collaborating, and whose values are realized. In that way you create an overview of possible effects, changes and interests. This teaches something about the conditions in which technology might support a ‘good practice’, while simultaneously evaluating the nature of this goodness.

We practice sniffer dog ethics in organizations that provide care for people with intellectual disabilities and experiment with new technologies. Caregivers try out promising technologies, and a committee helps them to analyse concerns about values, to anticipate possible problems and to develop promising possibilities. The sniffer dog anthropological ethicists sniff out the options, regard how people are juggling the emerging values, what they do with them, and to what effects this leads. In this way they can help to evaluate and shape complex practices.

## ‘Health’ in terms of everyday life values

Apart from sniffing out and experimenting with everyday values, there is another reason why an anthropology of everyday ethics may help medicine and health care. ‘Health’ contains many things. For example, at the start of the COVID-19 pandemic lockdowns, we studied what happened to vulnerable people, like people with psychiatric problems, intellectual disabilities, homeless people and elderly people living alone or in nursing homes. They had to stay inside, like everyone else. With academic colleagues and praticioners we were concerned that we had no idea how these people were doing when care was no longer given, and no one visited them at home. We wanted to know how they were doing. Thanks to our flexible methods we could rapidly bring a consortium together to keep an ear to the ground, through the telephone and through digital means.

One of the things we found, although not new but aggravated by the lockdowns, was the impact of loneliness on peoples’ lives. We revisited the numbers. More than 80 per cent of people with psychiatric problems was lonely. Elderly people were carried back to the times of occupation during WWII, where the outside world also posed a threat. Others died alone in a nursing home where they could no longer receive visitors. Some people collapsed due to lack of support and structure in their day, or because no other diseases seemed to exist other than COVID-19. There were people who did not understand anything of what was happening, other than that it was scary. People distrustful of authority were further isolated. Young people could not interact with each other, causing mental health problems that they have still not recovered from.

In short, loneliness, poverty, and policy effects on people’s health can co-determine if lives can be good. This makes it pivotal to learn what values are *simultaneously* at stake and which are not only part of the body. The COVID-19 lockdowns were unwanted experiments with social life, where social ties were taken away or strongly reduced. These experiments affected people’s health. Hence, medicine and health care need to look beyond their own disciplinary borders. And before one objects that health care is expensive enough as it is, I insist that we do not need to do *more* things, but have to do *different* things, things that are better tailored to the situation at hand. For instance, much more collaboration is possible in the public sector if we start seeing why that would be of value. Art can bring people together, can help to see new perspectives and experience things of value. Education can learn from the experiences of patients and caregivers. Patients may learn from patients. When *values* become central rather than costs alone, we will generate very different ideas about what we might do in relation to problems that do not go away. And once we know that, we can also establish what we are prepared to pay for it.

## Strengthening clinical practice by recognising everyday life values

The study of everyday values may strengthen clinical practice and people’s self-care by analysing how professionals and patients act upon what they think is important in the here and now. Medicine has a tradition of studying specificities through the clinical case history. Clinical case histories focus on individual patients. Knowledge about rare diseases is generated by studying specific instances of them, yet the clinical case study and its attention to specificity may be expanded. Not only individual patients can be the object of research, but also the use of certain technologies, the workings of certain values, specific research methods, ways of understanding problems, and so on. Type 1 diabetes is a problem of blood sugar regulation, but also a problem in everyday life. As we saw with the case study of Carola at the beginning of this article, insulin pumps might prioritize different types of values in living this everyday life compared to an insulin injection pen. We have to keep questioning our categories, the values that come along with them, and ask whether these are the important ones.

## Interdisciplinary knowledge about specificity

It is important to see that knowledge developed in the anthropology of everyday values does not result in generalizable knowledge. However, as with generalizable knowledge, specific results demand comparisons and careful considerations: why does this technique work *here*; what are the specificities of that hospital, or the situation of this patient, and how is that different to our setting? What do these differences mean for what we can and cannot do? To what kind of solutions does it lead *here*, and how might it work *there*?

This shows that implementing something new is always a process, and more complicated than simply handing the practice a tool for which it has been waiting. For example, the policy ideal that ‘technology’ is ‘the solution’ to make health care sustainable. When we do not specify *which* technology can lead to *what* kinds of solutions, and what forms of sustainability this might bring, technology will never make good on its promise. We then remain blind to the practical translations that are needed to make interventions useful and helpful. Technologies are not ‘finished’ things with inherent effects that are predictable when they reach a place where they are put into practice. They are further developed and made applicable ‘on the job’.^20^ This is a creative process that deserves far more attention than it gets now. What may work *here*, can work very differently *there*—or not at all.

The exchange between social scientific research and medical practice can be much improved. Because methods and epistemic traditions are so different, this exchange is difficult. Generalisable knowledge is very different from knowledge about specific situations. It needs to be treated and valued differently. Methodological diversity is an important concern in these times, and transdisciplinary collaboration is much needed for complex problems that do not go away. But this demands that we learn much more about the co-existence and value of different styles of knowing and forms of knowledge. To make Big Problems more specific.

## Conclusions

The anthropology of everyday ethics studies the specificity of everyday life values in their context, here, in health care practices for people with chronic disease. Rather than arguing in a prescriptive ethical register, such an approach studies the use and workings of values empirically. Different from quantitative studies, this research does not provide generalisable results, but provides insights in the *specificities* of contexts and situations and what becomes of importance there. The comparison of specificities allows for the analysis of questions as to what could be good to do given the particular context, or how certain interventions may be adapted to fit with what is deemed important locally. This fits with a clinical logic of handling the specific concerns for specific patients.

The empirical approach for the study of values brings different kinds of values to the fore, values that can not only be understood as *moral* in nature. The analysis of aesthetic values and the philosophical traditions related to their study, showed this. The analysis showed how a neo-liberal understanding of aesthetics as matters of taste created a blind spot for the social function of such values. Be it in questions about what is a good life for individuals, or in the styles of organising social and institutional lives, the social and conventional nature of what is deemed beautiful came to the fore. The implications point back to scientific practices, where what is true also relates to how methods creatively co-shape their objects of research, and simultaneously imply pathways for action. Different technical ways to manage bloodglucose levels bring along different values and possibilities for living with diabetes type I.

The term ‘everyday ethics’ is a descriptive term to denote practices in which values—or forms of the good—are actively addressed (strived for, cherished, proposed, achieved) and that actively shape what happens and what participants (people, words, things) do in certain situations. The notion of ethics here denotes an open conceptualisation of the good rather than a particular category of (moral) values. It mimics pre-modern understandings of morality that were open to different types of values. Everyday ethics may hence not be an ideal concept, given its association with a set of values that can be labelled as moral. I nonetheless wish to retain this concept to re-establish the connection with historical and present-day writings on everyday ethics. A more precise description would be, ‘Everyday ethics refers to forms of doing good-in-practice that include everyday forms of understanding and generating truth, beauty and other kinds of values’. This description emphasises what is done here, now, and what this implies for certain conceptualisations of the good in certain practices. These are neither prescriptive notions of the good, nor do they automatically lead to good practices.

The complexity of clinical practice is difficult or impossible to understand if everyday values in their context are not taken into account. Social scientific, ethnography-based research can help to support care practices. Not to blindly call for doing *more* or *fewer* things, but to zoom in and focus on what it might mean to do *other* things, things that fit a life that is as good as possible for as many people as possible.

## Footnotes

1 This resonates with the way in which Liszka (2021) has formulated a pragmatist ethics, which is an ethics that starts with identifying problems. However, here I want to stress that conceptualisations of what problems are, already include normative directions about how to do something about them.

2 Big Data research has relinquished the former virtue in quantitative research that it should be based on a hypothesis. The biggest problem I perceive with Big Data is that data are treated as having no context, as they are seemingly gathered to fit any kind of research question. We may become blind to what it is that we are actually measuring, and how this may not ‘add up’. See for instance Mol and Hardon (2020).

3 For examples of how to study ‘everyday ethics’, see Banks et al. (2013), Brodwin (2013), Banks and Brydon Miller (2018), and Banks (2020),

4 I take the open concept of ‘good’ from the work of Boltanski and Thévenot’s, which Thévenot (2001) dubbed the ‘sociology of the good’, which explores what people find important in different situations.

5 Everyday ethics may hence not be an ideal concept given its association with a set of values that can be labelled as moral. A more precise description would therefore be: ‘Everyday ethics refers to forms of doing good-in-practice that include everyday forms of truth, beauty and other kinds of values.’ This description emphasizes what is done *here, now*, and what this implies for particular conceptualizations of the good. These are neither prescriptive notions of the good nor do they automatically lead to good practices. I nonetheless wish to retain this concept to relate to work done on everyday ethics before (see above), and to re-evoke an ethics that is concerned with the variety of values in everyday life as well as their relationships.

6 The attention to things—objects—as actively participating in practices aimed at something good shows an inspiration from material semiotics, as developed in care studies and elsewhere (for examples see Mol 2008; Mol et al. 2010; Moser 2008; Winance 2007, 2010; Mol 2010; Willems 2010; Taylor 2010; Pols 2012, 2015; López-Gómez 2015; Skeide 2022; d’Hoop 2023).

7 In Pols (2013a, b) I describe how notions of dignity are understood either as a principle or refer to aesthetic values.

8 See Pols et al. (forthcoming 2024).

9 We organized the Festival of Unexpected Subjects about this theme: see Pols et al. (2023) for the essay about the use of creative methods in science, the artistic commentary on this through music and dance, and the participation of the audience.

10 See Pols (2013a, b).

11 This is the persistent message of science & technology studies (STS), see for instance Mol (2002).

12 Qualitative social science methods would do this trick. See our special issue in Medical Anthropology on methods to include subjects in research who are not verbally fluent, are cognitively diverse, or who do not speak the language of the researcher (Pols 2023a, b).

13 Where going abroad implies ‘making the strange common’, home-based ethographies have as their task to ‘make the common strange’. See Elliot (1994).

14 See Mol et al. 2010; Hout et al. 2015.

15 See Timmermans and Berg (2003).

16 See Dronkert (2023).

17 Anthony Kwame Appiah (2010) shows that ‘codes of honour’ keep practices alive for which rational arguments against these practices are already known by everyone. His examples are dueling, slavery or the binding of Chinese women’s feet.

18 Borry and colleagues (2005) described the ‘empirical turn in bioethics’, but remain hesitant about reconciling empirical research with ethics, which they maintain are two separate ways of working with the ‘is’ and the ‘ought’. In my contribution I attempt to integrate by grounding reasoning for the ought in empirical research, while empirically studying how people work to achieve something good, as moral actors and practical ethicists themselves. This paper mainly addresses the latter, but see Pols (forthcoming).

19 Fabrizio Turoldo (2024) reports on the debate between on ‘moral nose’, a concept that comes from the idea that moral problems are immediately and intuitively identified. This refers to what John Harris ironically calls the ‘“olfactory school of moral philosophy” (103) that identifies moral issues that lie “within sniffing distance” (103). Clearly, Harris does not like this type of moral philosophy very much. The sniffer dog ethics I discuss in this paper, however, do not refer to immediacy, but to a quest for identifying value concerns, good and bad.

20 We described four heuristics for getting to know these changes: both technologies and people can *unleash* one another, leading to new and unpredicted uses; or technologies and people can *tame* one another, leading to unpredicted hindrances. See Pols and Willems (2011).
